# High-throughput optimization of medium components and culture conditions for the efficient production of a lipopeptide pseudofactin by *Pseudomonas fluorescens* BD5

**DOI:** 10.1186/s12934-018-0968-x

**Published:** 2018-08-04

**Authors:** Piotr Biniarz, François Coutte, Frédérique Gancel, Marcin Łukaszewicz

**Affiliations:** 1 Department of Biotransformation, Faculty of Biotechnology, University of Wroclaw, Joliot-Curie 14a, 50-383 Wroclaw, Poland; 2 Univ. Lille, INRA, ISA, Univ. Artois, Univ. Littoral Côte d’Opale, EA 7394-ICV Institut Charles Viollette, 59000 Lille, France

**Keywords:** Biosurfactants, Lipopeptides, Pseudofactin, Production optimization, *Pseudomonas*, Design of experiments

## Abstract

**Background:**

Lipopeptides are a promising group of surface-active compounds of microbial origin (biosurfactants). These diverse molecules are produced mainly by *Bacillus* and *Pseudomonas* strains. Because of their attractive physiochemical and biological properties, biosurfactants are considered to be “green and versatile molecules of the future”. The main obstacles in widespread use of biosurfactants are mainly their low yields and high production costs. Pseudofactin (PF) is a lipopeptide produced by *Pseudomonas fluorescens* BD5. Recently, we identified two analogues, PF1 (C_16_-Val) and PF2 (C_16_-Leu), and reported that PF2 has good emulsification and foaming activities, as well as antibacterial, antifungal, anticancer, and antiadhesive properties. Reported production of PF in a mineral salt medium was approximately 10 mg/L.

**Results:**

Here, we report successful high-throughput optimization of culture medium and conditions for efficient PF production using *P. fluorescens* BD5. Compared with production in minimal medium, PF yield increased almost 120-fold, up to 1187 ± 13.0 mg/L. Using Plackett–Burman and central composite design methodologies we identified critical factors that are important for efficient PF production, mainly high glycerol concentration, supplementation with amino acids (leucine or valine) and complex additives (e.g. tryptone), as well as high culture aeration. We also detected the shift in a ratio of produced PF analogues in response to supplementation with different amino acids. Leucine strongly induces PF2 production, while valine addition supports PF1 production. We also reported the identification of two new PF analogues: PF3 (C_18_-Val) and PF4 (C_18_-Leu).

**Conclusions:**

Identification of critical culture parameters that are important for lipopeptide production and their high yields can result in reduction of the production costs of these molecules. This may lead to the industrial-scale production of biosurfactants and their widespread use. Moreover, we produced new lipopeptide pure analogues that can be used to investigate the relationship between the structure and biological activity of lipopeptides.

**Electronic supplementary material:**

The online version of this article (10.1186/s12934-018-0968-x) contains supplementary material, which is available to authorized users.

## Background

Biosurfactants (BS), a broad group of surface-active molecules, are secondary metabolites of microbial origin and they may have a variety of applications in industry, healthcare, or farming in the near future. Among the different classes of BS (e.g. glycolipids, lipopolysaccharides, phospholipids), lipopeptides (LPs) seem to be a particularly promising because of their compelling properties and high diversity. LPs can act as antibiotics, antiadhesives, antitumor compounds, cleaning, and foaming agents, but their action on living cells is not always clear. Potential applications of BS have been studied extensively and is the subject of many reviews and research publications [1–6]. Many bacterial and fungal species produce LPs. However, most of the published research on microbial LPs is performed using *Bacillus* sp. and *Pseudomonas* sp. [7–9].

To date, among bacterial LPs, only surfactin can be produced on a larger scale because of a long and successful process of optimization research, making its price more affordable [10, 11]. Other LP biosurfactants types such as iturin, fengycin, lichenysin, and mycosubtilin are still undergoing production optimization and scale-up outcome [5, 12–14]. Similarly, there are only a few commercially available high-purity LP standards, mainly surfactin from *Bacillus subtilis*, which is supplied by a few companies around the world. Besides surfactin, other LPs such as fengycin, iturin, lichenysin, and mycosubtilin are available. The least expensive is surfactin, with “industry-friendly” prices of approximately 300–500 €/kg, while others are much more expensive. This reflects the productivity and other difficulties of industrial-scale methods for the production and purification of LPs. Quality of such LPs will vary and may affect the range of potential applications, but in our opinion, these prices have already allowed the substantial growth of LP biosurfactant use in the pharmaceutical and food industries.

Although most efforts have been placed into research and commercialization of LPs of *Bacillus* origin, *Pseudomonas* sp. also produce a high diversity of LP families. According to the Norine database [9], there 365 peptides grouped into 39 families identified in the *Pseudomonas* genus. Unfortunately, no commercial *Pseudomonas*-origin LP standards are available. In contrast to *Pseudomonas* sp. LPs, different optimization and scaling-up approaches focused on *Pseudomonas* rhamnolipids [15], achieving industrially viable yields as high as 5 g/L [16], showing the potential of *Pseudomonas* sp. to produce valuable surface active compounds. Therefore, studies on optimizing production of LPs of *Pseudomonas* origin are of the great importance.

Pseudofactin (PF) is a LP that is produced by *Pseudomonas fluorescens* BD5 isolated from an Arctic Archipelago Svalbard [17]. Previously, two analogues of PF were identified: PF1 (C_16_-Gly-Ser-Thr-Leu-Leu-Ser-Leu-Val) and PF2 (C_16_-Gly-Ser-Thr-Leu-Leu-Ser-Leu-Leu). The only difference between these analogues is the amino acid in the eighth position. The PF peptide moiety is cyclized by the lactone bond between threonine and the eighth amino acid. PF exhibits antimicrobial [18, 19], antiadhesive [18, 20], and antibiofilm [18] activities against several pathogenic bacteria and yeast such as *Candida albicans*. PF antitumor activity has also been investigated [21]. Moreover, PF has better emulsifying properties compared to Tween 20 or Triton X-100 [17]. To date, only the properties of PF2 have been investigated [17]. *P. fluorescens* BD5, when cultivated in mineral salt medium (MSM), is capable of producing low amounts (< 10 mg/L) of PF, and it mainly produces PF2 [17]. Because of these low yields, it is currently impossible to test the industrial or pharmaceutical potential of PF. Therefore, PF production should be further optimized to provide affordable material for research.

While optimization of LP production has been undertaken by many laboratories around the globe, another direction in LP research is uncovering the LP structure–function relationship [22, 23]. To the best of our knowledge, all LPs are produced by microbes as mixtures of structural analogs that differ in length and/or branching of the hydrophobic chain as well as substitutions within the amino acid “head” of the molecules. For example, *B. subtilis* strains are capable of producing up to 12 surfactin analogs and *P. fluorescens* SS101 produces eight or more massetolide A isoforms [1, 5]. Even different LP families can be produced by one strain, as shown for different *B. subtilis* and *B. amyloliquefaciens* (*velezensis*) strains that produce several iturin, fengycin, and surfactin analogs [13, 24]. Proportions of LP analogs differ among bacterial strains and it can change in response to certain conditions, e.g. supplementation with different amino acids or carbon sources. This effect was probably best studied for *Bacillus.* For example, Liu et al. [25] showed that supplementation of culture medium with particular amino acids brings out production of surfactins with specified fatty acids. In this study Arg, Gln, or Val supplementation increased the amount of surfactin variants with even β-hydroxy fatty acids, while supplementation with Cys, His, Ile, Leu, Met, Ser, or Thr increased amount of surfactin variants with odd β-hydroxy fatty acids. Functions of different analogs have been poorly studied, but even a minor modification in the molecular structure can result in an extensive change in physiochemical properties [26, 27]. However, there are only few reports on identification of functions, properties, or possible applications of different LP analogues [26, 28, 29]. Structural analogues of a certain LP molecule can be used to study the relationship between their structure and biological activity. Such knowledge will improve the understanding of natural functions of LPs and will be also helpful in finding new applications for LPs or tailoring new molecules with a specific activity.

The aim of this work was to enhance the yield and study the variety of LPs produced by *P. fluorescens* BD5. We report successful high-throughput optimization of medium components and culture conditions for the efficient production of PF, using design of experiment (DoE) methodology, mainly Plackett–Burman and central composite design (CCD) models for planning the experiments. Moreover, we identified new PF isoforms (PF3 and PF4) in optimized cultures. We also reported a shift in the ratio of produced PF1 and PF2, in response to supplementation with different amino acids. According to our knowledge, no such a complex research has been performed for LPs of *Pseudomonas* origin.

## Results and discussion

Optimization of BS production requires a holistic approach that usually starts with a screening of efficient BS-producing strains (and/or strain engineering to produce BS effectively), followed by the finding the optimal bioprocess parameters (such as cultivation medium, temperature, oxygenation, and fermentation mode) and downstream processing (recovery and purification molecule of interest) [23, 30, 31].

In our work, we focused on the optimizing medium composition and culture conditions to maximize PF production, which is a LP produced by *P. fluorescens* BD5. We divided the optimization of PF production into three main stages. During the first stage, “Initial medium screening”, we tested 11 different media in standard Erlenmeyer shaken flasks to select the most promising medium to be further optimized. During the second stage, “High-throughput culture optimization”, we used the Biolector^®^ microfermentation system. This system allowed us to grow all cultures in high-throughput mode. In the Biolector^®^, we optimized medium that was previously selected during the “Initial medium screening”. We tested different additives to this medium (e.g. amino acids, nitrogen, or carbon sources) on bacterial growth and PF production. We also performed DoE-based experiments in the Biolector^®^ to identify the most significant factors influencing PF production. DoE methodology allowed us to establish a new medium with optimal levels of these significant factors. In the last stage, “Verification of optimized culture and further culture optimization”, we tested the optimal medium in different shaken Erlenmeyer flasks to investigate the influence of culture oxygenation and initial glycerol concentration on the bacterial growth and PF production. Moreover, we proposed a method for the production of specific PF structural analogues and identified new PF analogues.

### Initial medium screening

Different minimal media (e.g. MSM) are traditionally used to screen BS producers and to produce BS, using glucose or hydrocarbons as the sole carbon sources [1]. Also first protocols for the production of PF by *P. fluorescens* BD5 included stationary cultivation in MSM (supplemented with glucose) for 7 days, followed by clarification of cultures, extraction with ethyl acetate, and separation by RP-HPLC [17]. The method was simple, but time-consuming and inefficient. PF production under conditions mentioned above was estimated to be < 10 mg/L (data not shown) and required further optimization for the efficient PF production. During the initial screening we tested 11 different media (Table 6 and “Culture media” section). Some of these media were previously proposed and tested by other authors for the production of BS [23, 32–35] or the media were modified by us. Results are summarized in Table 1.

**Table 1:** Maximal growth (DCW), PF concentration (PF_C_), specific PF productivity (Y_P/X_), and pH during initial medium screening after 168 h of culture

Medium	Max. DCW (g/L)	Max. PF_C_ (mg/L)	Max. Y_P/X_ (mg/g)	Final pH
MSM	2.80 ± 0.13	10.1 ± 0.5	4.81 ± 0.28	4.1 ± 0.1
MSM +T	2.65 ± 0.24	11.8 ± 0.5	4.88 ± 0.58	4.0 ± 0.2
MSM +LB	3.89 ± 0.10	16.4 ± 0.3	5.04 ± 1.13	4.2 ± 0.2
MSM +G	2.46 ± 0.22	15.4 ± 1.0	6.51 ± 1.00	5.2 ± 0.1
MSM +G+LB	3.76 ± 0.06	20.9 ± 0.5	6.94 ± 0.65	5.3 ± 0.1
MSM +H	3.01 ± 0.38	4.2 ± 1.3	1.40 ± 0.46	6.6 ± 0.2
MSM +O	3.17 ± 0.63	4.7 ± 0.7	1.54 ± 0.24	6.7 ± 0.2
LB	10.10 ± 0.07	20.4 ± 0.6	2.37 ± 0.31	6.5 ± 0.1
KB	12.57 ± 0.09	80.8 ± 0.8	7.75 ± 0.25	7.1 ± 0.1
LA	7.31 ± 0.21	6.9 ± 0.8	1.48 ± 0.25	4.2 ± 0.1
LA +G	9.47 ± 0.18	23.0 ± 0.6	2.47 ± 0.08	6.5 ± 0.2

All results are shown as the mean value of three replicates ± SD. Culturing media are summarized in Table 6 and in “Culture media” section

PF yield obtained in MSM media was low, reaching a maximum of 20.9 ± 0.5 mg/L in MSM +G+LB medium. Additionally, in LB and LA media, PF_C_ reached only about 20 mg/L. We observed lower PF production in medium supplemented with glucose compared to medium containing glycerol (Table 1, *P *< 0.05). Low PF production in shaken cultures with glucose as a main carbon source can likely be explained by the low cell density in the cultures and by precipitation of LP at low pH and/or inhibition of LP production at low pH levels [6, 36]. With glucose as the carbon source, pH of the cultures decreased during the experiments, reaching 4.0–4.2 after the third culture day. In comparison, when glycerol was used (e.g. in MSM +G or LA +G), smaller changes in pH were observed (Table 1). The enrichment of minimal medium with complex additives (e.g. yeast extract or LB medium) caused higher PF production (Table 1, *P *< 0.05). Similar observations were reported earlier [37].

Supplementation of cultivation medium with hydrocarbons to produce BS is often mentioned for *Pseudomonas*, especially for rhamnolipid production [6, 38]. Therefore, we tested if addition of hexadecane or rapeseed oil will result in more efficient PF production. *P. fluorescens* BD5 is able to grow using hydrocarbons as a carbon source, but the PF yield drops to less than 5 mg/L (Table 1).

A modified LA medium, which is widely used to produce LP using *Bacillus* is a complex medium, enriched with growth-promoting yeast extract as well as LP production-promoting Glu. Modifying the traditional LA medium caused an increase in LP production by *B. subtilis* [33, 39]. Glucose is a main carbon source in LA medium (Table 6), and also in this case we observed a pH decrease in *P. fluorescens* BD5 cultures. Substitution of glucose with glycerol in LA +G medium resulted not only in maintaining more stable pH levels, but also in slightly better growth and in an almost threefold increase in PF yield (Table 1, *P *< 0.005). The beneficial effect of glycerol on surfactin production by *B. amyloliquefaciens (velezensis)* was recently demonstrated [24]. This suggests a positive role of glycerol, amino acids, and complex additives (yeast extract and/or peptone) in promoting growth and PF production by strain BD5.

*Pseudomonas fluorescens* BD5 was able to produce more PF (~ 80 mg/L), only when cultivated in KB medium (Table 1). KB medium was previously used to produce LP using *Pseudomonas* [40]. During culture in KB medium, we observed only minor changes in pH, the highest growth (maximum 12.57 ± 0.09 g/L DCW) the highest PF_C_ (80.8 ± 0.8 mg/L) and Y_P/X_ (7.75 ± 0.25 mg/g) among all tested media.

To test whether the culture oxygenation level may affect PF production, we additionally compared cultures in shaken flasks filled differently with media. We tested 20% (Table 1) and 40% filling volume (data not shown) and observed 10–20% higher growth and 10–50% higher PF yield (data not shown, *P *< 0.05) in cultures with a higher oxygenation level (lower filling volume). To compare different oxygenation levels, we calculated the theoretical OTR values in the cultures in Erlenmeyer flasks using equations proposed previously [41, 42]. OTR in the better oxygenated cultures (20% filling volume) was 41.7 mmol/L/h, whereas for 40% filling volume the OTR was approximately 1.8-times lower (23.5 mmol/L/h).

Initial screening results showed that, among tested media, KB was the best for the production of PF by *P. fluorescens* BD5. Moreover, culture oxygenation has a positive effect for both, microbial growth and PF production. Observed PF yield (in KB medium, 20% filling volume) increased approximately eightfold compared to cultures in MSM (Table 1).

### High-throughput culture optimization

Over the years, various supplements have been tested to improve LP production by bacteria and yeast. Generally, different carbon and/or nitrogen sources seem to be the most important and have been tested extensively [6]. Traditionally, these experiments are performed in various shaken flasks, which is not efficient. To perform these experiments in a high-throughput manner, we used the Biolector^®^ microfermentation system. The Biolector^®^ system was previously used to investigate the production of rhamnolipids [43] and LPs [33, 44].

During the initial experiments in the Biolector^®^, we tested the influence of different KB and KB-M1 media variants (Table 6 and “Culture media” section) and culture aeration on the growth and PF production by *P. fluorescens* BD5. The results are shown in Table 2.

**Table 2:** Maximal growth (DCW), PF concentration (PF_C_), and specific PF productivity (Y_P/X_) in the cultures during first medium screening in the Biolector^®^ after culture for 48 h

Medium	OTR = 30 mmol/L/h			OTR = 15 mmol/L/h		
	Max. DCW (g/L)	Max. PF_C_ (mg/L)	Max. Y_P/X_ (mg/g)	Max. DCW (g/L)	Max. PF_C_ (mg/L)	Max. Y_P/X_ (mg/g)
KB	5.66 ± 0.12	32.8 ± 1.3	7.77 ± 0.55	5.11 ± 0.35	28.6 ± 0.9	6.82 ± 0.14
KB-M1	5.17 ± 0.30	22.1 ± 0.9	5.03 ± 0.37	4.50 ± 0.15	18.7 ± 1.2	4.40 ± 0.37
KB-M1 +Leu	4.72 ± 0.43	57.9 ± 0.8	12.59 ± 0.41	4.51 ± 0.20	54.1 ± 0.5	12.38 ± 0.64
KB-M1 +Glu	5.25 ± 0.49	21.7 ± 1.3	6.46 ± 0.10	4.63 ± 0.26	20.0 ± 1.0	5.93 ± 0.24
KB-M1 +AA mix	3.97 ± 0.30	30.2 ± 0.8	8.18 ± 0.14	3.73 ± 0.37	28.8 ± 0.5	8.08 ± 0.27
KB-M1 +Am	4.25 ± 0.26	8.5 ± 0.9	2.02 ± 0.16	3.69 ± 0.49	7.6 ± 1.3	2.07 ± 0.35
KB-M1 +Leu+Am	4.76 ± 0.20	12.1 ± 1.0	2.68 ± 0.24	4.71 ± 0.07	10.2 ± 0.3	2.38 ± 0.14
KB-M1 +Glu+Am	4.95 ± 0.12	8.9 ± 1.3	2.02 ± 0.28	4.97 ± 0.23	8.1 ± 0.5	1.88 ± 0.05
KB-M1 +Cit	2.38 ± 0.39	2.4 ± 0.4	1.05 ± 0.22	2.47 ± 0.41	1.8 ± 1.0	0.72 ± 0.37
KB-M1 +Suc	3.32 ± 0.30	7.7 ± 0.2	2.72 ± 0.10	3.59 ± 0.18	3.2 ± 1.5	1.18 ± 0.52

All results are shown as the mean of three replicates ± SD. Culture media are summarized in Table 1 and in “Culture media” section

*OTR* oxygen transfer rate

During the first medium screening in the Biolector^®^, when *P. fluorescens* BD5 was cultured in KB medium, we observed that the maximal DCW was approximately 2.2–2.4-times lower and the maximal PF_C_ was approximately 2.4–2.8-times lower compared to cultures in Erlenmeyer flasks (see “Initial media screening” section). However, Y_P/X_ values for both cultures were similar (Tables 1 and 2). The OTR reached in Erlenmeyer flasks was 41.7 mmol/L/h, while in the Biolector^®^, the maximal OTR of 30 mmol/L/h was tested, which can explain observed differences in DCW and PF_C_.

The KB-M1 medium (Table 6) was design to test the effect of various additives for the growth and PF production by strain BD5 in the initial screening using the Biolector^®^. In this experiment, we tested the addition of nitrogen sources [amino acids: Leu, Glu, amino acid mixture, and (NH_4_)_2_SO_4_] as well as citrate and succinate as the sole carbon sources. We also investigated the impact of culture oxygenation. Our results suggest that for tested OTRs (30 and 15 mmol/L/h), there are no significant differences in the DCW (*P *> 0.120), but we were able to detect significantly larger amounts of PF in better oxygenated cultures (*P *< 0.05). Moreover, the addition of Leu has a strong positive effect on both, overall PF yield and specific PF productivity. The addition of 5 g/L Leu to KB-M1 medium caused a 2.6-fold increase in PF_C_ (*P *< 0.001) and 2.5-fold increase in Y_P/X_ (*P *< 0.001) with no influence on bacterial growth, compared to KB-M1 without Leu (Table 2). The positive influence of Leu on the production of LP production was previously shown for surfactin produced by *B. subtilis* [44]. Additionally, Glu may be another amino acid that has a positive effect on LP production [45, 46]. For *P. fluorescens* BD5, we detected no influence of Glu on bacterial growth and PF yield, but the Y_P/X_ slightly increased (*P *< 0.05) compared to the culture using KB-M1. We also tested the addition of an amino acid mixture (Leu, Ser, Thr and Gly), which was equivalent to the molar ratio of amino acids in the PF molecule (see “Culture media”), to produce PF. We detected a 1.3- to 1.5-fold increase in the PF yield (*P *< 0.01) as well as a 1.6- to 1.8-fold increase in Y_P/X_ (*P *< 0.05). This effect can likely be explained by the presence of Leu only. Addition of ammonium sulfate, which is commonly used as a nitrogen source for the production of BS [47] reduced PF production. Using citrate or succinate as a carbon source instead of glycerol also reduced growth and PF production (Table 2).

Similarly to Leu addition, complex additives seem to have a crucial impact on LP production. It was previously shown that culturing bacteria using different complex media affects the yield of LP and microbial growth [35]. Therefore, in the second round of Biolector^®^ screening, we tested six complex nitrogen additives (peptone, tryptone, proteose peptone, casitone, yeast extract, and soy peptone) to investigate their influence on bacterial growth, PF_C_, and Y_P/X_. The results are shown in Table 3.

**Table 3:** Maximal growth (DCW), PF concentration (PF_C_), and specific PF productivity (Y_P/X_) in cultures during testing of various complex nitrogen additives using the Biolector^®^ after culture for 66 h

Medium	Max. DCW (g/L)	Max. PF_C_ (mg/L)	Max. Y_P/X_ (mg/g)
KB-M2 +peptone	9.13 ± 0.31	514.0 ± 3.8	57.49 ± 1.27
KB-M2 +proteose peptone	6.01 ± 0.30	327.9 ± 11.7	54.98 ± 3.09
KB-M2 +yeast extract	21.86 ± 0.71	515.4 ± 10.7	23.86 ± 0.32
KB-M2 +tryptone	11.16 ± 0.33	628.7 ± 9.5	56.30 ± 1.89
KB-M2 +soy peptone	19.37 ± 0.48	77.6 ± 7.7	4.03 ± 0.23
KB-M2 +casitone	8.96 ± 0.44	397.2 ± 5.6	44.91 ± 1.68

All results are shown as the mean of three replicates ± SD. Culture media are summarized in Table 1 and in “Culture media” section

KB-M1 medium was further modified, which resulted in KB-M2 medium with higher glycerol and complex additive concentrations, as well as Leu addition (Table 6). Use of KB-M2 medium resulted in an intense microbial growth and increased the PF yield (Tables 2 and 3). During this Biolector^®^ run, we also used higher OTRs (50 mmol/L/h) for the better culture oxygenation.

The PF yield was high (> 300 mg/L) when five of the six of tested complex additives (peptone, proteose peptone, yeast extract, tryptone, and soy peptone) were used (Table 3). After 48 h of culture, PF yields were comparable for these additives (data not shown), and therefore we extended cultivation time to 66 h to detect possible differences.

Our results showed the importance of complex additives on DCW, PF_C_, and Y_P/X_. The observed maximal DCW values varied from approximately 6 g/L (proteose peptone) to approximately 22 g/L (yeast extract) and the maximal PF yield varied from approximately 80 mg/L (soy peptone) to approximately 630 mg/L (tryptone). The calculated maximal Y_P/X_ was the lowest in cultures containing soy peptone (approximately 4 mg/g) and highest (approximately 55–57.5 mg/g) in cultures containing peptone, proteose peptone, and tryptone (Table 3). The PF yield in KB-M2 +tryptone compared to MSM increased over 60-fold (Tables 1 and 3), and almost 20-fold compared to KB (Tables 2 and 3). We assumed that tryptone was the best complex additive for the PF production by *P. fluorescens* BD5.

DoE methodology is a powerful tool that can be used in bioprocess design and optimization. Different DoE approaches have been used to solve the complexity of BS production [10, 11, 33, 35, 48]. Therefore, we used a DoE strategy to further investigate the component concentration based on the previously defined media composition KB-M2 and also the effect of the aeration rate to efficiently produce PF by *P. fluorescens* BD5. The first model we implemented was 2^8^ × 3/6 Plackett–Burman screening. During these experiments, we tested the influence of eight factors (Table 4) on two responses (PF_C_ and Y_P/X_). Each factor was tested at one of three levels: low (−), medium (0), or high (+). The experimental design, factors’ levels, and the results are summarized in Table 4.

**Table 4:** Plackett–Burman screening design in the Biolector^®^

Variable levels	Variable (units)								Max. DCW (g/L)	Max. PF_C_ (mg/L)	Max. Y_P/X_ (mg/g)
	A	B	C	D	E	F	G	H			
	Glycerol (g/L)	Tryptone (g/L)	Leu (g/L)	MgSO_4_ (g/L)	Fe_2_(SO_4_)_3_ (mg/L)	K_2_HPO_4_ (g/L)	Trace el. (mg/L)	OTR (mmol/L/h)			
Low (−):	10	1	0.1	0.1	1	0.5	0.16	30			
Medium (0):	55	8	2	1	50.5	2.75	1.68	45			
High (+):	100	15	5	3	100	5	3.2	60			
*Experimental run*											
1	0	0	0	0	0	0	0	0	19.49 ± 0.23	134.2 ± 7.0	6.9 ± 0.4
2	−	−	−	−	−	−	−	−	4.81 ± 0.15	6.6 ± 0.6	1.4 ± 0.1
3	+	−	−	−	+	+	+	−	1.36 ± 0.06	4.3 ± 0.5	3.2 ± 0.3
4	+	−	+	−	−	−	+	+	4.61 ± 0.13	336.9 ± 7.0	73.2 ± 2.8
5	−	+	+	+	−	+	+	−	19.73 ± 0.42	55.0 ± 0.8	2.8 ± 0.1
6	−	−	+	+	+	−	+	+	11.17 ± 0.07	12.8 ± 0.7	1.1 ± 0.1
7	+	−	+	+	−	+	−	−	2.74 ± 0.04	129.4 ± 5.9	47.3 ± 2.3
8	0	0	0	0	0	0	0	0	19.49 ± 0.24	126.6 ± 7.2	6.5 ± 0.4
9	−	+	+	−	+	−	−	−	20.29 ± 0.18	132.7 ± 6.4	6.5 ± 0.3
10	−	+	−	−	−	+	+	+	16.19 ± 0.12	36.9 ± 0.7	2.3 ± 0.1
11	+	+	−	+	+	−	+	−	19.62 ± 0.19	412.4 ± 6.5	21.0 ± 0.3
12	+	+	+	−	+	+	−	+	23.14 ± 0.26	610.4 ± 5.9	26.4 ± 0.4
13	−	−	−	+	+	+	−	+	4.77 ± 0.07	1.0 ± 0.4	0.2 ± 0.1
14	+	+	−	+	−	−	−	+	18.66 ± 0.21	208.5 ± 6.5	11.2 ± 0.4
15	0	0	0	0	0	0	0	0	19.43 ± 0.38	111.8 ± 6.5	5.8 ± 0.3

Results for each experimental run are shown as the mean of three replicates ± SD. Maximal growth (DCW), PF concentration (PFC), specific PF productivity (Y_P/X_) after culture for 48 h. For further information on tested media, see “Culture media” section

**Fig. 1:**
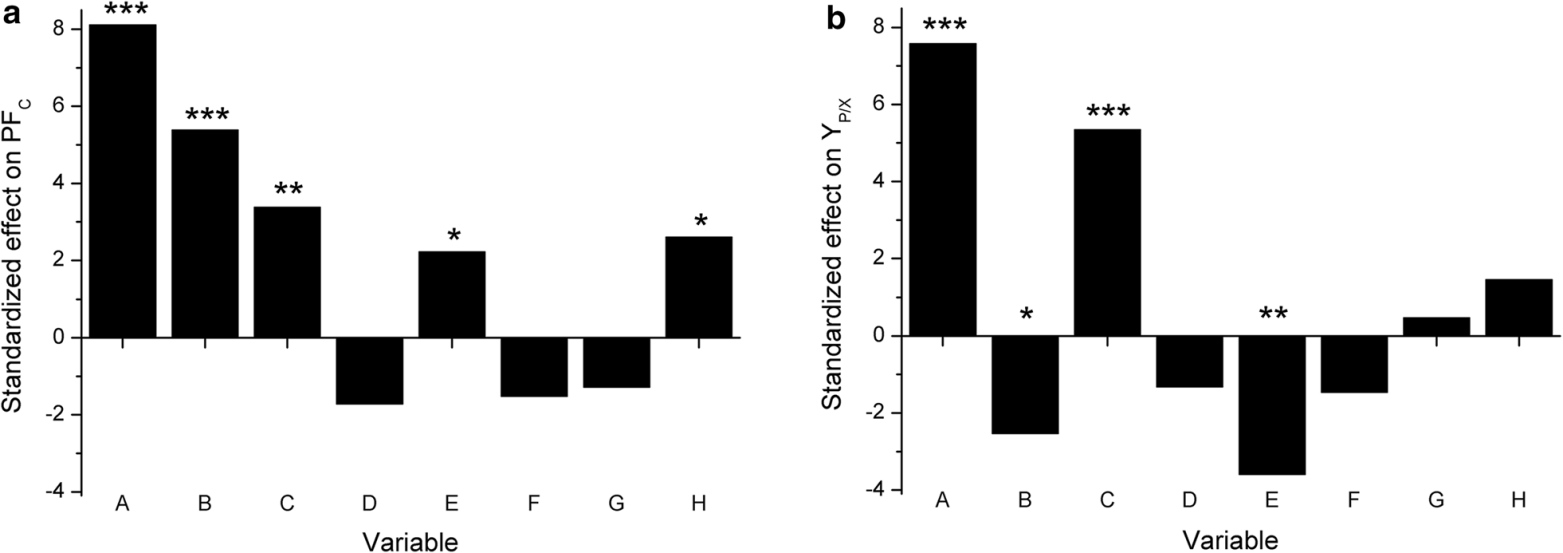
Standardized effects of the variables tested in the Plackett–Burman design in the Biolector^®^ on the responses: **a** shows the effect of variables on PF yield (PF_C_), and **b** shows the effect of variables on PF specific production (Y_P/X_). Variables: (A) glycerol, (B) tryptone, (C) Leu, (D) MgSO_4_, (E) Fe_2_(SO_4_)_3_, (F) K_2_HPO_4_, (G) trace elements, (H) OTR. Significance of effects (*P*-values) on the responses are shown for each variable: *P < 0.05, **P < 0.01, and ***P < 0.001

Both, maximal PF_C_ (610.4 ± 5.9 mg/L) and DCW (23.14 ± 0.26 g/L) were observed in run 12, whereas maximal Y_P/X_ (73.2 ± 2.8 mg/g) was observed in run 4. The lowest responses were observed in runs 2, 3, and 13, in which glycerol (except run 3), tryptone, and Leu were at low levels (Table 4). These suggest the importance of glycerol, tryptone, and Leu concentration, and culture oxygenation in PF production. To interpret the data and determine the influence of each of tested factor, the results were fitted to a linear function and analyzed using Statgraphics Centurion software. The standardized effect of each factor on the response was calculated and shown in Fig. 1, whereas detailed ANOVA analysis results are shown in Additional file 1: Table S1.

The Plackett–Burman screening design revealed the strong, positive effect of glycerol and Leu on both, PF_C_ and Y_P/X_. Moreover, tryptone had a strong positive effect on PF_C_, but a negative effect on Y_P/X_. This can be explained by the high growth-promoting activity of tryptone, which reflects on the Y_P/X_ values. The same situation was probably observed for Fe_2_(SO_4_)_3_ (Fig. 1 and Table 4). Culture oxygenation also seems to have a positive effect on PF_C_ and Y_P/X_, but for Y_P/X_, the relationship was insignificant (*P *= 0.156). Other tested variables (MgSO_4_, K_2_HPO_4_, and trace elements) had no significant effect on PF production in the tested conditions. This was surprising because trace elements (mainly Fe^2+^, Cu^2+^ and Mn^2+^ ions) are considered to be strong, positive inducers for BS production by *Bacillus* and *Pseudomonas*. However, mentioned effects were usually observed when Fe^2+^, Cu^2+^, and Mn^2+^ were added to minimal media [11, 49–52]. In our case (rich media with high concentrations of complex nutrients), it is likely that no further supplementation with trace elements was required (Fig. 1). Supplementation with another divalent cation (Mg^2+^) was also shown to be LP-promoting [53]. Additionally, phosphates were shown to enhance LP production [35]. The opposite effect can be observed for PF production, but these effects are not significant (*P *= 0.138 for PF_C_ and *P *= 0.154 for Y_P/X_).

The significance of the model was investigated using Fisher’s F-test (Additional file 1: Table S1). The *r*^*2*^ values for the model were 0.786 and 0.766 for PF_C_ and Y_P/X_, respectively. The relatively low *r*^*2*^ could be explained by the interactions between variables, but the model shows an acceptable level of fit [33].

The three most significant variables (glycerol, tryptone, and Leu) from the Plackett–Burman screening (Fig. 1) were chosen for further optimization using a CCD design in Biolector^®^. CCD was used to build a second-order (quadric) model of the impact of variables on the tested responses (PF_C_ and Y_P/X_) and to investigate potential relationships between variables. We used the CCD 2^3^ + star (rotatable and orthogonal) model with 23 runs, including nine centerpoints. Each variable was tested at three levels (low, medium, and high), but two star-points (low and high) were also added. For all experimental runs DCW and PF_C_ were measured and Y_P/X_ was calculated. The experimental design, factors’ levels and the results are summarized in Table 5.

**Table 5:** CCD experimental design in the Biolector^®^

Variable levels	Variable (g/L)			Max. DCW (g/L)	Max. PF_C_ (mg/L)	Max. Y_P/X_ (mg/g)
	A	B	C			
	Glycerol	Tryptone	Leu			
Low star point (− α):	16	3.9	0.8			
Low (−):	50	8.0	2.5			
Medium (0):	100	14.0	5.0			
High (+):	150	20.0	7.5			
High star point (+ α):	184	24.1	9.2			
*Experimental run*						
1	0	− α	0	9.43 ± 0.14	224.3 ± 13.3	23.8 ± 1.3
2	0	0	0	15.41 ± 0.22	397.9 ± 16.4	25.8 ± 1.3
3	0	0	0	15.74 ± 0.25	411.6 ± 14.6	26.1 ± 1.0
4	0	0	0	16.05 ± 0.30	425.1 ± 7.5	26.5 ± 0.6
5	− α	0	0	29.64 ± 0.80	200.2 ± 10.1	9.5 ± 0.5
6	−	−	−	22.28 ± 0.37	137.9 ± 14.2	6.2 ± 0.7
7	0	0	− α	16.70 ± 0.14	236.6 ± 12.7	14.2 ± 0.8
8	0	0	0	16.78 ± 0.19	396.2 ± 9.5	23.6 ± 0.6
9	0	+ α	0	20.01 ± 0.38	382.5 ± 10.7	19.1 ± 0.4
10	−	+	−	30.93 ± 0.79	189.6 ± 12.7	6.1 ± 0.4
11	+	+	+	0.42 ± 0.03	34.9 ± 6.5	82.7 ± 19.4
12	−	+	+	27.94 ± 0.41	264.0 ± 6.3	9.4 ± 0.2
13	0	0	0	15.33 ± 0.31	412.2 ± 9.6	26.9 ± 0.8
14	0	0	0	16.23 ± 0.33	392.0 ± 8.4	24.2 ± 0.6
15	+ α	0	0	0.01 ± 0.00	4.4 ± 1.0	723.6 ± 82.8
16	0	0	0	16.34 ± 0.28	405.6 ± 11.8	24.8 ± 0.8
17	+	−	−	0.74 ± 0.05	19.1 ± 3.2	25.9 ± 5.0
18	0	0	+ α	15.27 ± 0.29	640.2 ± 5.2	41.9 ± 0.8
19	0	0	0	16.68 ± 0.45	405.8 ± 6.7	24.3 ± 0.9
20	0	0	0	15.95 ± 1.09	409.9 ± 12.3	25.7 ± 2.1
21	+	−	+	0.14 ± 0.02	9.5 ± 2.4	68.0 ± 24.1
22	+	+	−	0.18 ± 0.02	21.5 ± 3.5	122.3 ± 22.3
23	−	−	+	25.44 ± 0.32	438.2 ± 13.0	17.2 ± 0.6

Results for each experimental run are shown as the mean value of three replicates ± SD: maximal growth (DCW), PF concentration (PFC), specific PF productivity (Y_P/X_) after culture for 48 h. For further information on the tested media, see “Culture media” section. The OTR for the cultures was set at 55 mmol/L/h

Maximal PF_C_ of 640.2 ± 5.2 mg/L was observed in run 18, when high Leu concentration (star point) was added (9.2 g/L) and glycerol and tryptone were used at the medium level. This suggests a strong, positive effect of Leu supplementation on PF production (Tables 4, 5 and Fig. 1). The inhibition of bacterial growth and PF production can be also easily identified for cultures with > 100 g/L glycerol. This can be explained by the osmotic stress on cells and/or insufficient oxygen supply in the cultures. Y_P/X_ can be misleading because high values have been calculated for runs 11, 15, 17, 21, and 22, resulting from low DCW (Table 5). Therefore, all data was used to model the effect of variables on PF_C_ and data from runs 11, 15, 17, 21, and 22 was omitted in analyzing the effect on Y_P/X_. The obtained cubic regression equations describe the dependence of the variables (glycerol, tryptone and Leu concentrations) and responses (PF_C_ and Y_P/X_):

$$\hat{P}F_{C} = - \;757.394 + 9.978 \times A + 53.634 \times B + 117.834 \times C - 0.0545 \times A^{2} + 0.066 \times AB - 0.381 \times AC - 1.787 \times B^{2} - 1.610 \times BC - 2.655 \times C^{2}$$

$$\hat{Y}_{P/X} = - \;47.394 + 0.733 \times A + 2.868 \times B + 6.778 \times C - 0.004 \times A^{2} + 0.004 \times AB - 0.014 \times AC - 0.109 \times B^{2} - 0.071 \times BC - 0.257 \times C^{2}$$

where ${\hat{\rm{P}}}{{\rm{F}}_{\rm{c}}}$ is the predicted PF concentration and Ŷ_P/X_ is the predicted PF specific productivity. Variables in the equation are identical to those shown in Table 5: (A) glycerol (g/L), (B) tryptone (g/L), and (C) Leu (g/L). Optimal levels of variables calculated for the CCD model and the medium compositions were found to be as follows:

- For maximizing PF_C_: (A) 67 g/L of glycerol, (B) 12.1 g/L of tryptone, and (C) 9.2 g/L of Leu. This medium was named KB-Opt-CCD (Table 6).
- For maximizing Y_P/X_: (A) 82 g/L of glycerol, (B) 11.5 g/L of tryptone, and (C) 9.2 g/L of Leu.

**Fig. 2:**
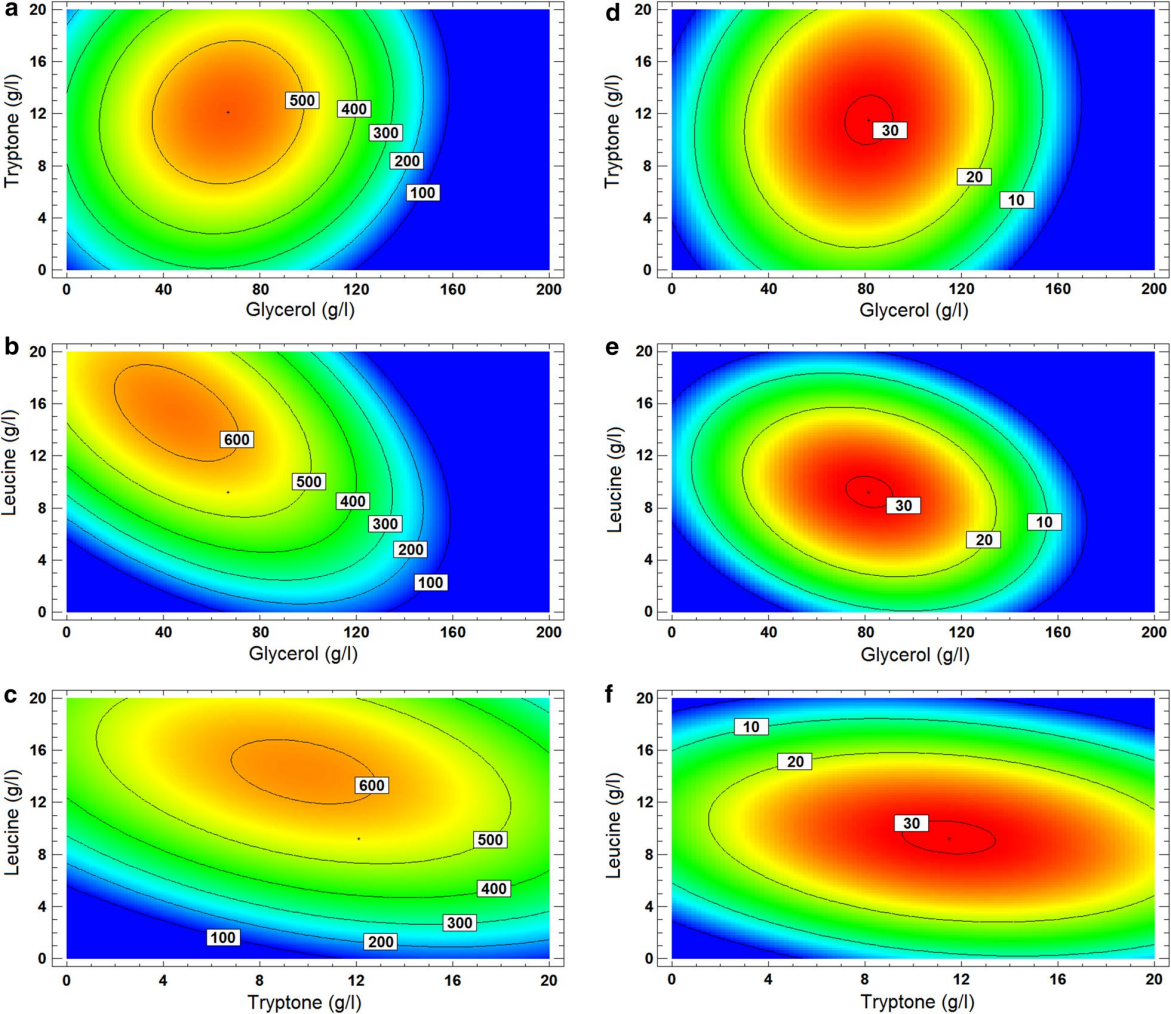
Contour plots of the estimated response for PF_C_ and Y_P/X_. Estimated PF_C_ (mg/L) and Y_P/X_ (mg/g DCW) values are shown as color patterns (cold to hot), as isolines and in white boxes. **a** Contour plot of PF_C_ for glycerol and tryptone. Leu is held at the CCD-modelled optimal level of 9.2 g/L. **b** Contour plot of PF_C_ for glycerol and Leu. Tryptone is held at CCD-modelled optimal level of 12.1 g/L. **c** Contour plot of PF_C_ for tryptone and Leu. The glycerol level is held at the CCD-modelled optimal level of 67 g/L. **d** Contour plot of Y_P/X_ for glycerol and tryptone. Leu is held at the CCD-modelled optimal level of 9.2 g/L. **e** Contour plot of Y_P/X_ for glycerol and Leu. Tryptone is held at the CCD-modelled optimal level of 11.5 g/L. **f** Contour plot of Y_P/X_ for tryptone and Leu. The glycerol level is held at the CCD-modelled optimal level of 8.2 g/L

Maximized predicted PF_C_ and Y_P/X_ values were 551.6 mg/L and 30.4 mg/g, respectively, and are lower than the maximal observed responses (640.2 ± 5.2 mg/L and 41.9 ± 0.8 mg/g, cf. Table 5). The significance of the model was investigated using Fisher’s F-test (Additional file 1: Table S2). The *r*^*2*^ shows acceptable values for the model fit of 0.846 and 0.781 for PF_C_ and Y_P/X_, respectively. The model predictions of PF_C_ and Y_P/X_ are shown on contour plots (Fig. 2).

### Verification of optimized cultures and further culture optimization

For the verification of high-throughput optimization and for further optimization of culture conditions and medium components, we performed cultures in KB-Opt +Leu medium in shaken flasks. KB-Opt was derived from KB-Opt-CCD by increasing the concentration of tryptone to 15 g/L and Leu to 10 g/L (Table 6 and “Culture media” section), which simplified media preparation. We detected no significant differences in growth and PF production kinetics for BD5 cultured in KB-Opt and KB-Opt-CCD in shaken flasks when the same glycerol concentration was used (data not shown).

**Fig. 3:**
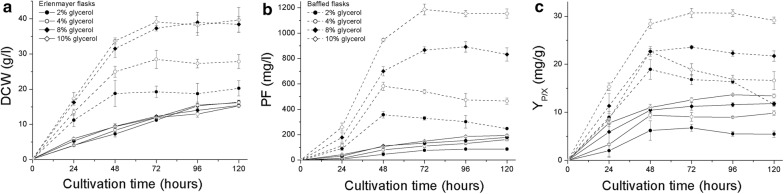
Growth curves (**a**), PF production kinetics (**b**) and specific PF production kinetics (**c**) of *P. fluorescens* BD5 grown in KB-Opt +Leu medium with various initial glycerol concentrations and in different shaken flasks. Results for each data point are shown as the mean value of three replicates ± SD. For further information on media, see “Culture media” section. Solid lines, Erlenmeyer flasks; dashed lines, baffled flasks

We tested the influence of the initial glycerol concentration (20, 40, 80, and 100 g/L) on growth and PF production in 300-mL Erlenmeyer flasks (20% filling volume) and 500-mL baffled flasks (20% filling volume). This resulted in different oxygenation of the cultures and carbon/nitrogen ratios. The growth curves and PF production kinetics are shown in Fig. 3.

The oxygenation of the cultures seems to have critical impact on both, cell growth and PF production. For *P. fluorescens* cultured in 300-mL Erlenmeyer flasks, the growth rate was similar independently from initial glycerol concentration (0.153–0.168 g/h between 0 and 72 h of cultures). Additionally, no logarithmic growth phase was observed (Fig. 3a). Maximal PF_C_ in the Erlenmeyer flasks ranged from 86.1 ± 7.0 mg/L (20 g/L glycerol) to 194.1 ± 7.0 mg/L (100 g/L glycerol) whereas Y_P/X_ from 6.80 ± 0.71 to 14.35 ± 0.11 mg/g. Here, approximately 20-fold increase in PF yield can be observed compared to MSM medium and an approximately 2.5-fold increase compared to KB medium (Table 1 and Fig. 3b). For Y_P/X_, a threefold and 1.8-fold increase was observed compared to MSM and KB media, respectively. The theoretical OTR calculated for these cultures was 47.8 mmol/L/h [41, 42].

In the next step of verification of the optimal media, we tested baffled Erlenmeyer flasks (500 mL, 20% filling volume) to test if the cultures can be better supplied with oxygen. According to Gupta and Rao (2003), calculated K_L_a values for the cultures in a baffled flask should be approximately doubled in comparison to the cultures performed in the same medium and filling volume using Erlenmeyer flasks without baffles. Therefore, the calculated theoretical OTR for the cultures in baffled Erlenmeyer flasks was estimated to be 81.1 mmol/L/h [41, 42, 54]. The growth rates of these cultures were higher (0.387–0.698 g/h between 0 and 48 h of cultures) and maximal DCW reached almost 40 g/L for the cultures with 80 and 100 g/L glycerol (Fig. 3a). The maximal observed PF_C_ reached 1187.0 ± 13.0 mg/L, when 100 g/L glycerol was used in a baffled flask. Additionally, Y_P/X_ was the highest in the described conditions and reached 30.35 ± 0.98 mg/g. Considering the KB-Opt 100 g/L glycerol medium, use of baffled flasks increased DCW 2.6-fold, PF_C_ 5.9-fold, and Y_P/X_ 2.3-fold compared to standard Erlenmeyer flasks. This shows the importance of oxygen transfer in bacterial cultures, especially when rich media are used.

### Production and identification of PF analogues

LPs are usually produced by bacteria as a mixture of analogues. The differences between analogues include length and/or branching of hydrophobic moiety, as well as substitutions in amino acids in a peptide part [1]. It was previously shown that changes in culture conditions and/or medium composition can result in a shift in the relative abundance of LP analogues produced by *B. subtilis* [45, 55]. To the best of our knowledge, no such influence was shown for *Pseudomonas* sp.

**Fig. 4:**
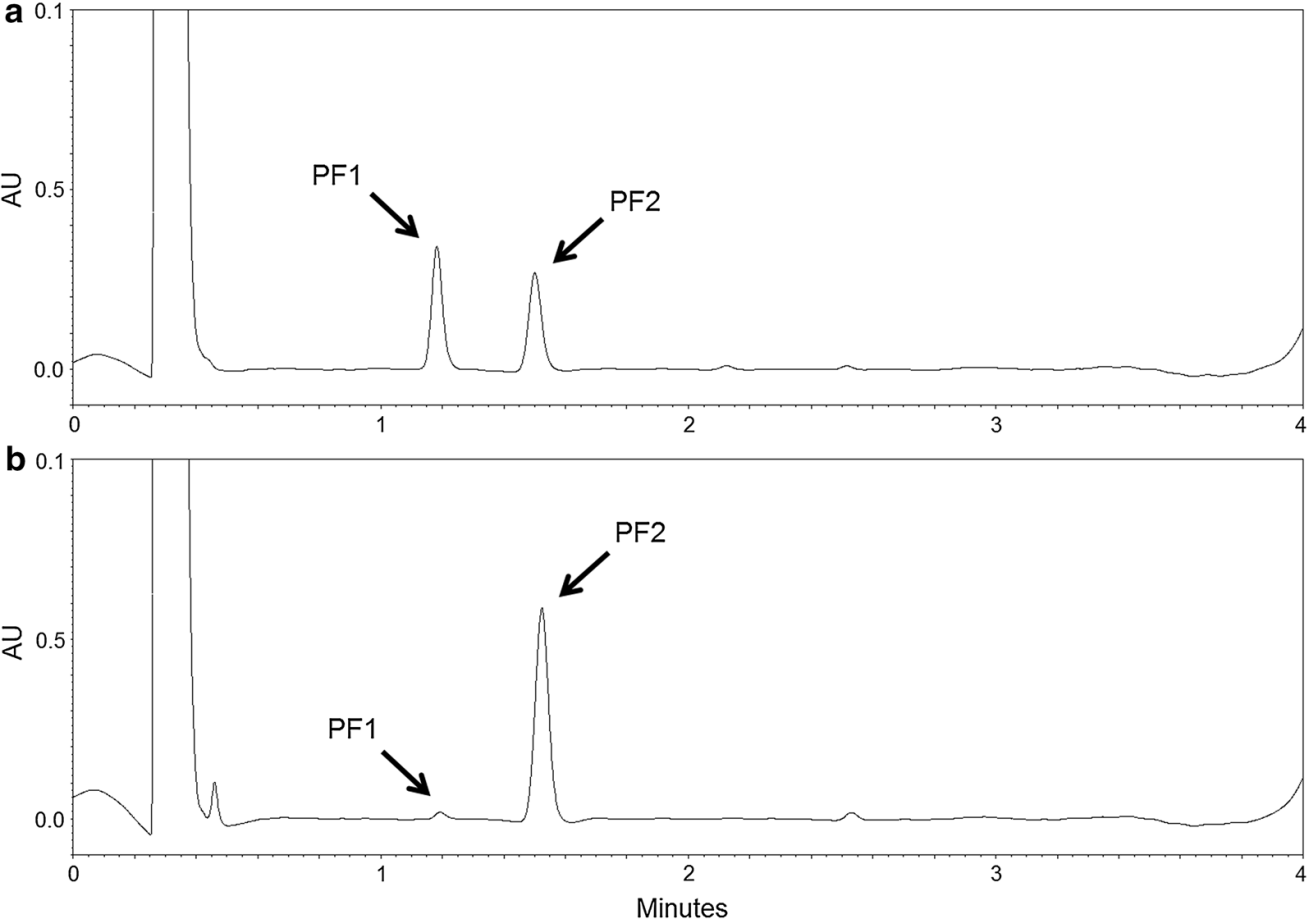
HPLC chromatograms of cell-free culture supernatants from *P. fluorescens* BD5 cultured in KB-Opt +Val (**a**) and KB-Opt +Leu (**b**) media. PF1 and PF2 peaks are marked

We tested if the supplementation of KB-Opt medium (Table 6) with different amino acids will result in the increase of PF yield or in the shift in relative abundance of PF analogues. Supplementation of KB-Opt medium with 10 g/L Leu (KB-Opt +Leu) resulted in the increased PF2 production (C_16_-Leu). Here, the relative abundance of analogues was 2.3 ± 0.6% PF1 and 97.7 ± 4.7% PF2, with the total PF_C_ of 1173.3 ± 23.9 mg/L (Fig. 4b), while for KB-Opt medium supplemented with 10 g/L Val (KB-Opt +Val), production of PF1 (C_16_-Val) emerged. Relative abundance of PF analogues was 53.0 ± 3.3% PF1 and 47.0 ± 0.2% PF2, with the total PF_C_ 1114.1 ± 17.4 mg/L (Fig. 4a). HPLC chromatograms of KB-Opt (supplemented with Val or Leu) cell-free supernatants are shown in Fig. 4.

**Fig. 5:**
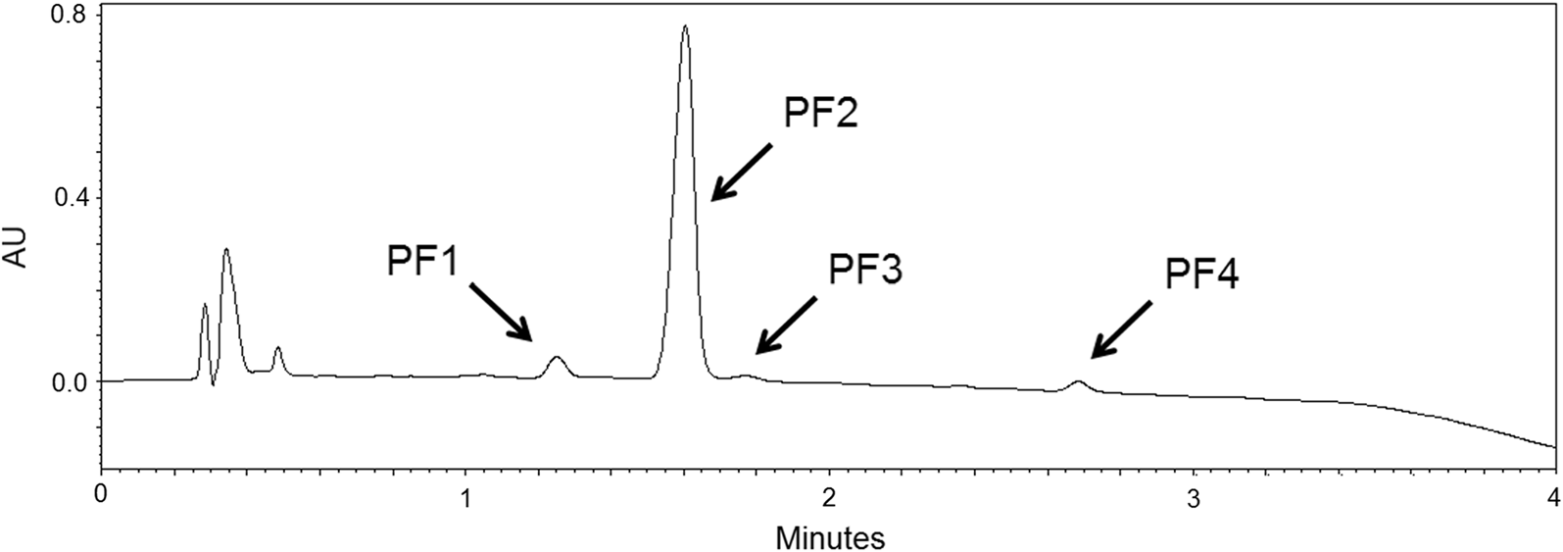
HPLC chromatogram of the SPE extract of the cell-free culture supernatants from *P. fluorescens* BD5 cultured in KB-Opt-Leu. PF1, PF2, PF3, and PF4 peaks are marked

During the experiments, as the total PF yield increased, we identified trace quantities of new PF analogues. These newly identified compounds were called PF3 (C_18_-Val) and PF4 (C_18_-Leu) and the production of these analogues by strain BD5 emerged using KB-Opt-Leu or KB-Opt-Val media (Table 6) and a longer incubation time (up to 7 days). PF3 and PF4 were identified in SPE extracts of cell-free supernatants from *P. fluorescens* BD5 cultures (Fig. 5), and they were analyzed using UPLC-MS/MS, as described before [32]. The 1053 and 1067 [H^+^] m/z adducts were assigned to the newly identified peaks.

**Fig. 6:**
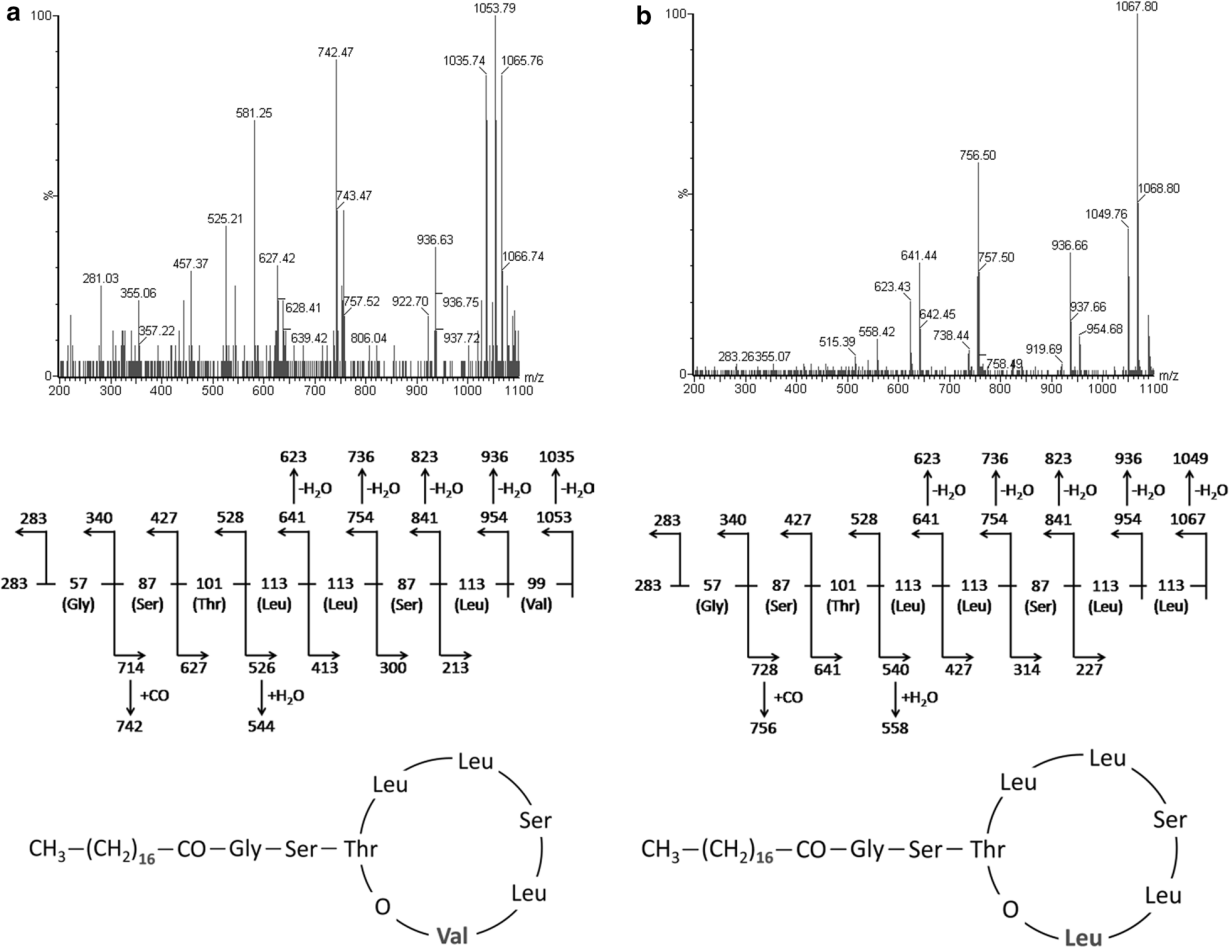
TOF-MS/MS spectra (top panel) of PF3 (**a**) and PF4 (**b**), with structure elucidation (middle panel). Ions at 1053 and 1067 m/z were used as precursors for analyses. Bottom panel: proposed structures of PF3 (**a**) and PF4 (**b**), based on Janek et al. [17]

The 1053 and 1067 m/z ions were used as the precursor ions for TOF–MS/MS (Time of flight MS/MS) analyses of PF3 and PF4, respectively. PF1 and PF2 TOF–MS/MS data and sequence elucidation were used for comparison [17]. TOF–MS/MS data and proposed structures of PF3 and PF4 are shown in Fig. 6.

We measured PF analogues concentrations during cultivation of *P. fluorescens* BD5 in KB-Opt-Leu medium (Table 6), using previously established HPLC methods [32]. Under the described conditions, strain BD5 is capable of producing 8.8 ± 0.6 mg/L of PF3 and 20.1 ± 0.7 mg/L of PF4 in a total PF_C_ of 1082.9 ± 24.5 mg/L. PF analogues can be further produced and individually purified using semi-preparative HPLC. Then, physiochemical and biological properties of analogues can be investigated, which may lead to a better understanding of the LP structure–function relationship.

## Conclusions

- Three-step optimization of medium components and culture conditions for the efficient production of a lipopeptide pseudofactin (PF) was performed.
- Critical factors, substantial for efficient production of PF have been identified, including high glycerol concentration (up to 100 g/L), amino acid (leucine or valine), and complex additive (e.g. tryptone) supplementation as well as high culture aeration.
- Optimized King’s B medium (KB-Opt) for the efficient production of PF was described. Up to 1200 mg/L of PF can be produced by *P. fluorescens* BD5, which is almost 120-fold increase compared with the cultures in MSM.
- The ratio between PF structural analogues produced by *P. fluorescens* BD5 can be controlled by a simple amino acid supplementation. Supplementation of optimized medium with leucine increases the production of PF2 (C_16_-Leu), whereas supplementation with valine enhances the production of PF1 (C_16_-Val).
- Two new analogues of PF were identified: PF3 (C_18_-Val) and PF4 (C_18_-Leu). These analogues were identified in cultures with a high initial glycerol concentration.

## Methods

### Chemicals

Chemicals and media components were purchased from manufacturers as follows: yeast extract, proteose peptone, tryptone, casitone (pancreatic digest of casein), and soy peptone, (Becton–Dickinson, USA); peptone (Difco, USA); glucose, K_2_HPO_4_, MgSO_4_, (NH_4_)_2_SO_4_, KCl, NaCl, Fe_2_(SO_4_)_3_, MnSO_4_, CuSO_4_, and sodium citrate (POCH, Poland); sodium succinate (Avantor, USA); LB, MOPS, l-leucine (Leu), l-valine (Val), l-glutamic acid (Glu), glycine (Gly), l-serine (Ser), and l-threonine (Thr) (Bioshop, Canada); glycerol (VWR International, USA); hexadecane (Sigma-Aldrich, Germany); and rapeseed oil (ZT Kruszwica S.A., Poland).

### Culture media

Various media tested for the production of PF are summarized in Table 6. Before starting the culture, the pH of all media used was set at 7.0 with 6 M NaOH or 6 M HCl. All media were buffered with 100 mM MOPS (3-(*N*-morpholino)propanesulfonic acid).

**Table 6:** Media used to test PF production by *P. fluorescens* BD5

Medium		Medium composition	Medium application	References
KB	King’s B	10 g/L glycerol, 20 g/L proteose peptone, 1.5 g/L K_2_HPO_4_, 1.5 g/L MgSO_4_, 100 mM MOPS	Initial media screening, high-throughput optimization	[59]
KB-M1	Modified King’s B M1	10 g/L glycerol, 2 g/L proteose peptone, 1.5 g/L K_2_HPO_4_, 1.5 g/L MgSO_4_, 100 mM MOPS, various modifications were added (see “Culture media” section)	High-throughput optimization	This work
KB-M2	Modified King’s B M2	40 g/L glycerol, 5 g/L complex additive, 5 g/L Leu, 1.5 g/L K_2_HPO_4_, 1.5 g/L MgSO_4_, 100 mM MOPS, various complex additives were added (see “Culture media” section)	High-throughput optimization	This work
KB-Opt-CCD	Optimized King’s B (CCD model)	67 g/L glycerol, 12.1 g/L tryptone, 9.2 g/L Leu, 0.5 g/L K_2_HPO_4_, 0.1 g/L MgSO_4_, 50 mg/L Fe_2_(SO_4_)_3_, 100 mM MOPS	High-throughput optimization, further culture optimization	This work
KB-Opt	Optimized King’s B	20–100 g/L glycerol, 15 g/L tryptone, 10 g/L Leu/Val, 0.5 g/L K_2_HPO_4_, 0.1 g/L MgSO_4_, 50 mg/L Fe_2_(SO_4_)_3_, 100 mM MOPS	Further culture optimization, production of PF analogues	This work
LA	Modified Landy’s	20 g/L glucose, 2.3 g/L (NH_4_)_2_SO_4_, 2 g/L Glu, 0.5 g/L MgSO_4_, 1.6 mg/L CuSO_4_, 1.2 mg/L Fe_2_(SO_4_)_3_, 0.4 mg/L MnSO_4_, 1 g/L yeast extract, 100 mM MOPS	Initial media screening	Modified from [39]
LB	Lysogeny broth	10 g/L tryptone, 5 g/L yeast extract, 10 g/L NaCl	Seed cultures, initial media screening	–
MSM	Mineral salt medium	20 g/L glucose, 2.3 g/L (NH_4_)_2_SO_4_, 0.5 g/L sodium citrate, 2 g/L K_2_HPO_4_, 0.1 g/L MgSO_4_, 100 mM MOPS	Initial media screening	Modified from [17]

For further information see “Culture media” section

*MOPS * 3-(*N*-morpholino)propanesulfonic acid, *Leu* l-leucine, *Glu* l-glutamic acid, *Val* l-valine

When stated, media were modified to test the influence of various nitrogen and/or carbon sources on bacterial growth. Basic media compositions are shown in Table 6, whereas various media modifications are described below.

During the initial medium screening, four basal media (MSM, LB, KB, and LA) and seven modified basal media were tested (Tables 1 and 6) to choose medium for further optimization experiments. Basal media were modified to investigate the impact of the carbon source and adding nitrogen or trace elements on bacterial growth and PF production. Modifications to the MSM medium (Tables 1 and 6) are as follows: MSM +LB (1/25 v/v LB added), MSM +G (10 g/L glycerol added instead of glucose), MSM +G+LB (10 g/L glycerol added instead of glucose and 1/25 v/v LB added), MSM +T (trace elements added: 2.4 mg/L Fe_2_(SO_4_)_3_, 1.6 mg/L CuSO_4_, and 0.4 mg/L MnSO_4_), MSM +H (10 g/L hexadecane added instead of glucose), and MSM +O (10 g/L rapeseed oil added instead of glucose). We also tested the change from glucose to glycerol (10 g/L) in LA medium (which resulted in LA +G medium).

KB medium was selected to be further investigated and modified in the Biolector^®^ experiments. During the first round of Biolector^®^ screening, we tested the impact of different nitrogen additives (selected amino acids and (NH_4_)_2_SO_4_) and carbon sources (citric acid and succinic acid) on bacterial growth and PF production. This resulted in KB-M1 medium with the following modifications: +Leu (5 g/L Leu added), +Glu (5 g/L Glu added), +AA mix (5 g/L of a mixture of four amino acids Leu, Ser, Thr, and Gly in molar ratio 4:2:1:1 added), +Am (5 g/L (NH_4_)_2_SO_4_ added), +Leu+Am (2.5 g/L Leu and 2.5 g/L (NH_4_)_2_SO_4_ added), +Glu+Am (2.5 g/L Glu and 2.5 g/L (NH_4_)_2_SO_4_ added), +Cit (10.6 g/L citric acid added instead of glycerol), and +Suc (9.7 g/L succinic acid added instead of glycerol).

During the second round of Biolector^®^ screening, we investigated the influence of different complex additives on bacterial growth and PF production. This resulted in KB-M2 medium with the following complex additives (5 g/L): peptone, tryptone, proteose peptone, casitone, yeast extract, or soy peptone.

#### Plackett–Burman screening in the Biolector^®^

The Plackett–Burman design was used to screen critical factors that are important for PF production. The following eight factors were tested: concentration of seven medium components (A. glycerol, B. tryptone, C. Leu, D. MgSO_4_, E. Fe_2_(SO_4_)_3_, F. K_2_HPO_4_, G. trace elements: MnSO_4_ and CuSO_4_), and culture oxygenation (H. OTR). MOPS concentration was maintained at 100 mM. Variable levels and the Plackett–Burman design summary are shown in Table 4.

#### CCD screening in Biolector^®^

The CCD design was used to optimize the medium composition for maximizing PF yield. Analysis of the Plackett–Burman design allowed us to choose three factors to be further optimized using the CCD model, which were: A. glycerol, B. tryptone, and C. Leu. Concentrations of other medium components were as follows: 0.1 g/L MgSO_4_, 0.5 g/L K_2_HPO_4_, 50 mg/L Fe_2_(SO_4_)_3_, and 100 mM MOPS. The OTR was set at 55 mmol/L/h. Variable levels and the CCD design are shown in Table 5. Analysis of DoE data allowed us to establish the KB-Opt-CCD medium, which was further tested for the production of PF in shaken flasks.

#### Further culture optimization

The influence of the initial glycerol concentration in KB-Opt medium for the PF production was tested. KB-Opt was derived from KB-Opt-CCD. Initial concentrations of 20, 40, 80, and 100 g/L of glycerol were tested. Leucine (10 g/L) was added to the medium. For the production of PF analogues, the following modifications to the KB-Opt medium were used: +Leu (10 g/L Leu added) or +Val (10 g/L Val added).

### Strain used and preculture

The arctic isolate *P. fluorescens* BD5 (PCM B/00115) was used to produce PF [17]. The BD5 strain was preserved as glycerol stock (− 80 °C) in the Department of Biotransformation, Faculty of Biotechnology, University of Wrocław, Poland, and it was grown on LB agar plates at 28 °C. After 48-hours of incubation, single colonies were used to inoculate 10 mL of LB medium. These precultures were grown overnight (18–22 h) at 28 °C with agitation (160 rpm). Next, the bacteria were pelleted (15 min, 10,000*g*), washed twice with 0.9% NaCl and resuspended in 5 mL of 0.9% NaCl. The OD at 600 nm (OD) was measured, and these suspensions were used to inoculate cultures for PF production.

### Initial media screening

Initial medium screening experiments were performed in 300-mL Erlenmeyer flasks (Schott DURAN), using 20% or 40% filling volumes. Media used for the cultivation of microorganisms are summarized in Table 6 and in “Culture media” section. Cultures were inoculated with an overnight seed culture, to reach the final OD of 0.01, and then incubated at 28 °C with agitation (160 rpm). Every 24 h, samples were aseptically taken to measure biomass (OD) and the PF concentration (“Analytical methods” section). Cultures were incubated for a maximum of 7 days. Each experiment was repeated at least three times and measurements of each variable were performed in triplicate.

### High-throughput culture optimization

High-throughput screening of medium components and culture conditions, which are important for PF production, was performed in 48-well flower microplates in the microfermentation systems Biolector^®^ (m2p-labs GmbH, Germany), which is available on the REALCAT platform at the University of Lille (France) and in the Department of Biotransformation, Faculty of Biotechnology, University of Wrocław (Poland). Media used for the cultivation of microorganisms are summarized in “Culture media” section and in Table 6. Cultures were inoculated with an overnight seed culture to the final OD of 0.01, and then stored at 28 °C and agitated. Certain oxygen transfer rates (OTRs) were reached by applying different working volumes (from 800 µL to 2 mL) and agitation speeds as stated by the Biolector^®^ manufacturer [56]. In each experiment, microbial growth (scattered light), pH, and pO_2_ were monitored in real time, while PF was measured in a few time points for each experiment. Scattered light measurements were calibrated against dry biomass and OD measurements (“Analytical methods” section). Cultures were incubated for a maximum of 48 h. Each experiment was repeated at least three times and measurements of each variable were performed in triplicate.

### Verification of optimized cultures and further culture optimization

Verification of optimized medium and further culture optimization were performed in various shaken flasks to obtain different aerations of the cultures using 300-mL Erlenmeyer flasks (Schott DURAN, 20% filling volume) and 500-mL baffled Erlenmeyer flasks (Schott DURAN, 20% filling volume). Here, KB-Opt +Leu medium was used (Table 6 and “Culture media” section). Different concentrations of glycerol, ranging from 20 to 100 g/L were tested. Cultures were inoculated with an overnight seed culture, to reach the final OD of 0.1, and then incubated at 28 °C with agitation (180 rpm). Cultures were incubated for a maximum of 5 days. At certain time points, samples were aseptically taken for biomass (OD) and PF concentration analyses (“Analytical methods” section). Each experiment was repeated at least three times and measurements of each variable were performed in triplicate.

### Production and identification of PF analogues

PF analogues were produced in KB-Opt medium with modifications (Table 6 and “Culture media” section). Cultures were performed in 500-mL baffled Erlenmeyer flasks (Schott DURAN, 20% filling volume). The KB-Opt medium was supplemented with Val (10 g/L) or Leu (10 g/L) for the production of PF1 and PF2, respectively. High glycerol concentration in KB-Opt (up to 100 g/L) and longer incubation time (up to 7 days) were used to increase the PF3 and PF4 yields. Cultures were inoculated with an overnight seed culture, to reach the final OD of 0.1, and then incubated at 28 °C with agitation (180 rpm). At certain time points, samples were aseptically taken for biomass (OD) and PF concentration analyses (“Analytical methods” section). Total PF and single analogue concentrations were measured using RP-HPLC (reversed phase high-performance liquid chromatography). PF analogues were identified using RP-UPLC-MS/MS (reversed phase ultra-performance liquid chromatography mass spectrometry) (“Analytical methods” section). Each experiment was repeated at least three times and measurements of each variable were performed in triplicate.

### Analytical methods

Biomass concentration (dry cell weight, DCW) was evaluated by measuring the optical density (OD) at 600 nm using an Oddyssey DR/2500 spectrophotometer (Hach, USA). OD was correlated with dry biomass measurements (data not shown). When OD measurements were not possible (e.g. because of medium emulsification when rapeseed oil or hexadecane were used as a carbon source), DCW was measured directly using the method proposed by Greenberg [57].

PF concentration (PF_C_) was measured using HPLC in cell-free culture supernatants, as described previously [32]. Two HPLC systems were used: system 1 consisted of a Beckman Gold 126 Pump and a Knauer Variable Wavelength Monitor equipped with a Macherey–Nagel C18 Isis column (50 mm × 4.6 mm, 1.8 μm) under the control of LP-Chrom software (Lipopharm, Poland). The column was kept at room temperature during analyses. System 2 consisted of Waters Acquity Arc Quaternary Solvent Manager-R, Sample Manager FTN-R and 2489 UV/VIS Detector, equipped with a Waters Cortecs C18 column (50 mm × 4.6 mm, 2.7 µm). The column was maintained at 40 °C during the analyses. Samples were diluted ten times with methanol before analyses. Areas of all detected PF peaks were summed and compared to the PF standard curve, as reported previously [32].

To calculate the specific PF yield (Y_P/X_), the PF_C_ was divided by DCW (data from the same time points). Y_P/X_ is expressed as the number of PF in mg produced per 1 g of DCW (mg/g).

UPLC-MS/MS analyses of PF analogues were performed using an Waters Acquity UPLC System with a 2996 PDA detector and a Waters Xevo QTof MS System, as described previously [32]. Here, Waters Acquity BEH C18 column (100 mm × 2.1 mm, 1.7 μm) at 40 °C was used. MS analyses were conducted as follows: positive mode ESI; source temperature, 150 °C; desolvation gas temperature, 350 °C; desolvation gas flow, 800 L/h; cone gas flow 20 L/h; cone voltage, 10 V; and capillary voltage 3 kV. The samples were analyzed in the range of 800–1200 *m/z*. Next, certain parameters were changed and the m/z 1053.79 and 1067.81 ions were used as precursor ions for MS/MS analyses as follows: cone voltage, 40 V; capillary voltage, 3 kV; and collision energy, 40 V. The ions were analyzed in the range of 200–1100 *m/z*.

K_L_a (volumetric gas–liquid mass transfer coefficient) for the cultures in shaken flasks was calculated using the equation proposed by Fahim et al. [41]. OTR (oxygen transfer rate) in shaken flasks was calculated using the equation proposed by Funke et al. [42]. To calculate OTR values, we assumed, that maximal oxygen solubility in culture medium is 0.2031 mmol/L [58].

### Data analysis

Microsoft Excel software was used to analyze the obtained data. Means, standard deviations (SD), and relative standard deviations (RSD) were calculated. Statgraphics Centurion XVII (Statpoints Technologies Inc., USA) was used for designing Plackett–Burman and Central Composite Design (CCD) experiments, as well as for data analysis. Statistical analyses were also performed with Microsoft Excel software using a paired *t*-test with Bonferroni correction. *P* values of < 0.05 were considered significant.

## Additional file

**Additional file 1: Table S1.** Analysis of variance for the influence of tested variables on the responses in the Plackett–Burman screening design in the Biolector®. **Table S2.** Estimated effects on tested variables and analysis of variance for the cubic CCD model on PF yield (PF_C_) and PF specific production (Y_P/X_).
