# Supplementary material for: High-throughput optimization of medium components and culture conditions for the efficient production of a lipopeptide pseudofactin by Pseudomonas fluorescens BD5
Source: Microb Cell Fact. 2018 Aug 4;17:121. doi: 10.1186/s12934-018-0968-x (PMC6076405; doi:10.1186/s12934-018-0968-x)
Supplement: Supplementary file 1 — Additional file 1: Table S1. Analysis of variance for the influence of tested variables on the responses in the Plackett–Burman screening design in the Biolector®. Table S2. Estimated effects on tested variables and analysis of variance for the cubic CCD model on PF yield (PFC) and PF specific production (YP/X). [file 12934_2018_968_MOESM1_ESM.docx]

**Additional file 1**

**High-throughput optimization of medium components and culture conditions for the efficient production of a lipopeptide pseudofactin by *Pseudomonas fluorescens* BD5**

Piotr Biniarz^1^, François Coutte^2^, Frédérique Gancel^2^, Marcin Łukaszewicz^1,^*

^1^Department of Biotransformation, Faculty of Biotechnology, University of Wroclaw, Joliot-Curie 14a, Wroclaw 50-383, Poland

^2^Univ. Lille, INRA, ISA, Univ. Artois, Univ. Littoral Côte d’Opale, EA 7394-ICV Institut Charles Viollette, F-59000 Lille, France

^*^ Corresponding author

Email: marcin.lukaszewicz@uwr.edu.pl, telephone: +48713756250

Table S1. Analysis of variance for the influence of tested variables on the responses in the Plackett–Burman screening design in the Biolector®.

| **Source** | ***Df*** | **PF_C_** | | **Y_P/X_** | |
| --- | --- | --- | --- | --- | --- |
|  |  | **Sum of squares** | ***F*-ratio** | **Sum of squares** | ***F*-ratio** |
| **A:** Glycerol | 1 | 530639.0 | 65.89 | 7042.0 | 57.41 |
| **B:** Tryptone | 1 | 232758.0 | 28.90 | 786.8 | 6.41 |
| **C:** Leu | 1 | 92264.1 | 11.46 | 3486.9 | 28.43 |
| **D:** MgSO_4_ | 1 | 23813.6 | 2.96 | 214.6 | 1.75 |
| **E:** Fe_2_(SO_4_)_3_ | 1 | 40073.4 | 4.98 | 1580.1 | 12.88 |
| **F:** K_2_HPO_4_ | 1 | 18582.2 | 2.31 | 259.8 | 2.12 |
| **G:** Trace elements | 1 | 13259.5 | 1.65 | 28.3 | 0.23 |
| **H:** OTR | 1 | 54327.8 | 6.75 | 257.6 | 2.10 |
| Blocks | 2 | 2.56711 | 0.00 | 0.4 | 0.00 |
| Total error | 34 | 273828.0 |  | 4170.4 |  |
| Total (corr.) | 44 | 1279550.0 |  | 17826.8 |  |

Responses: PF yield (PFC) and PF specific production (YP/X).

Table S2. Estimated effects on tested variables and analysis of variance for the cubic CCD model on PF yield (PF_C_) and PF specific production (Y_P/X_).

| **Source** | ***Df*** | **PF_C_** | | | | | **Y_P/X_** | | | | |
| --- | --- | --- | --- | --- | --- | --- | --- | --- | --- | --- | --- |
|  |  | **Estimated**  **effect** | **Standard**  **error** | **Sum of**  **squares** | ***F*-ratio** | ***P*-value** | **Estimated**  **effect** | **Standard**  **error** | **Sum of**  **squares** | ***F*-ratio** | ***P*-value** |
| Model | 9 | 409.16 | 18.5233 |  |  | 0.000 | 25.59 | 1.4423 |  |  | 0.000 |
| **A:** Glycerol | 1 | -189.02 | 30.0939 | 243962 | 39.45 | 0.000 | -7.60 | 2.3433 | 394.1 | 10.51 | 0.003 |
| **B:** Tryptone | 1 | 26.46 | 30.0939 | 4780 | 0.77 | 0.385 | -2.17 | 2.3433 | 32.01 | 0.86 | 0.361 |
| **C:** Leu | 1 | 153.41 | 30.0939 | 160711 | 25.99 | 0.000 | 8.92 | 2.3433 | 542.89 | 14.48 | 0.000 |
| **AA** | 1 | -272.40 | 27.8997 | 589521 | 95.33 | 0.000 | -19.69 | 2.1724 | 3080.72 | 82.16 | 0.000 |
| **AB** | 1 | 39.90 | 39.3196 | 6368 | 1.03 | 0.317 | 2.22 | 3.0616 | 19.80 | 0.53 | 0.472 |
| **AC** | 1 | -95.15 | 39.3196 | 36214 | 5.86 | 0.021 | -3.58 | 3.0616 | 51.12 | 1.36 | 0.251 |
| **BB** | 1 | -128.65 | 27.8997 | 131487 | 21.26 | 0.000 | -7.88 | 2.1724 | 493.73 | 13.17 | 0.001 |
| **BC** | 1 | -48.30 | 39.3196 | 9332 | 1.51 | 0.228 | -2.12 | 3.0616 | 18.06 | 0.48 | 0.492 |
| **CC** | 1 | -33.19 | 27.8998 | 8751 | 1.42 | 0.242 | -3.22 | 2.1724 | 82.19 | 2.19 | 0.148 |
| Total error | 35 |  |  | 216444 |  |  |  |  | 1312.30 |  |  |
| Total (corr.) | 45 |  |  | 1402370 |  |  |  |  | 6000.75 |  |  |
